# Uncovering microbial dynamics in soil: a reproducible metagenomics pipeline for fertilization studies

**DOI:** 10.1093/bib/bbaf631.017

**Published:** 2025-12-12

**Authors:** Dorra Rjaibi, Alia Benkahla, Oussama Souiai

**Affiliations:** Laboratory of Bioinformatics Biomathematics and Biostatistics, LR16IPT09, Institut Pasteur de Tunis, University of Tunis El Manar, Tunis, Tunisia; Laboratory of Bioinformatics Biomathematics and Biostatistics, LR16IPT09, Institut Pasteur de Tunis, University of Tunis El Manar, Tunis, Tunisia; Laboratory of Bioinformatics Biomathematics and Biostatistics, LR16IPT09, Institut Pasteur de Tunis, University of Tunis El Manar, Tunis, Tunisia

## Abstract

**Aim:**

Soil microbial communities play pivotal roles in nutrient cycling, soil fertility, and sustainable crop production, yet reproducible metagenomic analyses remain elusive due to methodological inconsistencies, impeding multi-omics integration. This study develops a robust, reproducible Snakemake-based pipeline to elucidate microbial taxonomic and functional shifts in response to diverse fertilization regimes, fostering insights into ecosystem resilience and agricultural sustainability.

**Methods:**

We engineered a modular Snakemake workflow for automated, scalable processing of soil metagenomes, emphasizing long-read sequencing data. Key modules encompass quality control of long reads, host contaminant removal, taxonomic classification via advanced algorithms, differential abundance analysis, and functional pathway annotation. Applied to soil samples from bio-compost and chemical fertilizer treatments, the pipeline facilitates network modeling and ecological inference, while its design supports seamless extension to transcriptomic and metabolomic layers for holistic soil–plant-microbe investigations.

**Results:**

Pipeline application unveiled pronounced microbial restructuring: bio-compost amplified diversity in beneficial taxa linked to nitrogen fixation and plant growth promotion, contrasting with chemical fertilizers that favored stress-resistant specialists and altered metabolic profiles toward xenobiotic degradation. Network analyses exposed enhanced cooperative interactions under organic inputs, with functional annotations tying microbial genes to improved nutrient bioavailability and reduced soil degradation, validated across replicated datasets for statistical rigor.

**Conclusion:**

This standardized pipeline, enhanced by long-read sequencing, enables reproducible soil microbiome exploration, bridging microbial ecology and agricultural applications. It lays a foundation for multi-omics integration, supporting sustainable fertilization strategies and advancing soil health understanding.

